# No Spillover Effect of the Foreclosure Crisis on Weight Change: The Diabetes Study of Northern California (DISTANCE)

**DOI:** 10.1371/journal.pone.0151334

**Published:** 2016-03-17

**Authors:** Janelle Downing, Andrew Karter, Hector Rodriguez, William H. Dow, Nancy Adler, Dean Schillinger, Margaret Warton, Barbara Laraia

**Affiliations:** 1 School of Public Health, University of California, Berkeley, California, United States of America; 2 Division of Research, Kaiser Permanente, Oakland, California, United States of America; 3 Department of Psychiatry, University of California San Francisco, San Francisco, California, United States of America; 4 Department of Medicine, University of California San Francisco, San Francisco, California, United States of America; University of Westminster, UNITED KINGDOM

## Abstract

The emerging body of research suggests the unprecedented increase in housing foreclosures and unemployment between 2007 and 2009 had detrimental effects on health. Using data from electronic health records of 105,919 patients with diabetes in Northern California, this study examined how increases in foreclosure rates from 2006 to 2010 affected weight change. We anticipated that two of the pathways that explain how the spike in foreclosure rates affects weight gain—increasing stress and declining salutary health behaviors- would be acute in a population with diabetes because of metabolic sensitivity to stressors and health behaviors. Controlling for unemployment, housing prices, temporal trends, and time-invariant confounders with individual fixed effects, we found no evidence of an association between the foreclosure rate in each patient's census block of residence and body mass index. Our results suggest, although more than half of the population was exposed to at least one foreclosure within their census block, the foreclosure crisis did not independently impact weight change.

## Introduction

The decline of the U.S. economy between 2007 and 2009 was the largest since the Great Depression. The housing market collapse, later known as the foreclosure crisis, contributed to the rise in unemployment during the Great Recession [1, 2]. Mortgage default has often been precipitated by job loss [3]. Despite the interdependence between the housing and labor markets, research on economic recessions and health has often omitted foreclosures from the discussion.

An emergent body of research on foreclosures and health includes several studies on the health of individual homeowners in response to experiencing a foreclosure [4–13]. Research has demonstrated the financial spillover effects of a foreclosure in a neighborhood [14, 15], but only a small number of studies of the spillover effects of foreclosures on health exist [16–21]. These studies have yielded evidence suggesting that residing in a high foreclosure environment may contribute to poor health.

Only a single study has previously examined the relationship between BMI and residing near a foreclosure [20]. The authors found that living within 100 meters of a foreclosure was associated with a 0.2 units increase in BMI. This finding is counter to the evidence from the unemployment literature; excess weight fell during economic downturns in the United States [22]. Our study contributes to this literature by examining how the neighborhood foreclosure rate relates to individual body mass index (BMI) among an insured population with a chronic disease (diabetes) in California.

To further develop our hypotheses, we borrowed and built upon theories propounded in the recessions and health literature [23] to inform the pathways by which the neighborhood foreclosure rate may affect individual BMI. First, a neighboring foreclosed home may act as a chronic stressor for homeowners who anticipate or experience a decline in the value of their home. A fall in housing equity could trigger default or reduce resources available to purchase inputs for health. In addition, homeowners or renters may experience or anticipate higher rates of crime or social disorganization in their neighborhood as homeowners vacate [24–33].

Next, an increase in the foreclosure rate of a community can reduce the tax base [34] and thus reduce local governments resources [35] devoted to health-promoting activities. Foreclosures might erode social capital within a community if there is high attrition of socially connected residents, resulting in poorer health for those remaining [36]. Finally, high foreclosure rates in the region could lead to job loss, particularly for those employed in construction-related industries.

People with diabetes are particularly sensitive to stressors, which can decrease insulin sensitivity [37], and this increased stress, coupled with reductions in wealth, neighborhood health-promoting activities, and social cohesion could add to the challenges in diabetes self-management and healthy eating [38, 39]. Given the existing evidence and theory, we expected to observe a net positive relationship between foreclosure rate and BMI in our study population.

To inform our prediction about how health insurance status might influence our results, we drew from on a study by Currie which found that an additional foreclosure in a given zip-code increased non-elective hospital visits (including those for preventable diabetes-related complications) among those with public, but not private health, insurance [17]. In our population, we expected that poorer self-management of BMI would be larger among those with Medicaid insurance. In addition, we stratified by additional demographic characteristics.

To test our hypotheses, we linked public foreclosure deed records from 2006 to 2009 at the census block level to clinical records of Kaiser Permanente patients with diabetes who lived in one of nine Bay Area counties. We used an individual-fixed effects approach, adjusting for the unemployment rate, housing prices, and time trends, to estimate the effect of the census block foreclosure rate on mean annual BMI.

## Materials and Methods

### Study Design and Subjects

The clinical data was obtained from electronic health records of patients with diabetes receiving uniform access to care within a large, integrated, healthcare delivery system Kaiser Permanente Northern California (KPNC) from 2007 to 2011. Patients with type 1 or unknown type diabetes, histories of lower extremity amputation or pregnancy or cancer diagnosis within the study period and 1-year prior were excluded. Patients were retained for analysis if they had at a geocodable address record for at least two years over the period from 2006 to 2009 in one of nine Bay Area counties. An additional 5.2% percent had only one BMI measure over all four years and were excluded from the analysis.

Foreclosure data included address-level residential deed and housing price data compiled by DataQuick [40] from the nine Bay Area counties between 2006 and 2009. Data on area-level demographics were also collected from the American Community Survey (ACS) [41]. Data from the Local Area Unemployment Statistics database were used for county-level unemployment rates [42]. Additional individual-level data was based on a sub-set of patients (n = 8,923) within our population who had completed the Diabetes Study of Northern California (DISTANCE) survey at baseline, in 2006 [43]. The Institutional Review Boards of Kaiser Permanente Northern California and University of California, Berkeley approved this study. Clinical records were anonymized and de-identified prior to analysis.

### Health outcome

The main outcome for this study, body mass index (BMI, kg/m^2^), was an annual average of all measurements during patient ambulatory visits from 2007 to 2010. The mean number of BMI measurements per patient was 1.3 per year and 8.1 over the four-year period.

### Neighborhood Foreclosure Measures

We define foreclosure as the event in which the residential deed is transferred to the new owner. Our data did not differentiate between properties that were transferred back to the bank (real-estate owned) and those sold at auction. We used ArcGIS 10 and Data Scientist Toolkit in R to assign geographic coordinates to the foreclosures. Only 19 (<1%) of the foreclosure addresses not geocodable.

The primary exposure of interest was the annual foreclosure rate within the patient’s census block. The number of foreclosures was divided by the number of housing units in each block using data from the US Census 2000. In this region, the length of an average block is 100m, the distance at which a foreclosure is expected to have economic spillover effects on the values of neighboring properties [15].

### Covariates

Time-varying neighborhood-level covariates from 2006 to 2009 were used to control for area-level variation. We adjusted for mean housing prices at the zip-code level and county unemployment rate. Additional block-level variables were created using the American Community Survey (ACS) including population-density, proportion of the population under the federal poverty line, proportion of owner-occupied units, and proportion of the population identified as White.

The following individual-level time-varying covariates were collected from 2007 to 2010. Medicaid status (1/0) was included as a proxy for change in income or employment status. We included a Charlson comorbidity index score [44], which predicts the ten year mortality for a patient with a range of conditions, and annual indicators for use of medications clinically associated with weight change (insulin, oral diabetes medications, and psychiatric medications such as selective serotonin reuptake inhibitors (SSRIs) that can cause weight gain or weight loss.)

For the subsample with DISTANCE survey responses, we created measures of income (continuous), non-housing wealth (1 = $10k+, 0 = less than $10k), education (0 = graduate degree+, 1 = less than high school diploma, 2 = high school diploma, 3 = bachelors degree), employment status (1 = employed full-time, 0 = not employed), and partnership status (1 = married or co-habiting, 0 = non-married, divorced, separated, widowed). For the same subsample, we created homeownership status (1 = owner, 0 = renter) included individuals without missing data on housing status. Individual homeownership status was coded as missing for who reported living “rent-free” (i.e. with a relative) or moved at least once from 2007 to 2009 because we were unable to determine if they rented or owned for the entire period.

### Statistical Methods

To examine the relationship between foreclosures and BMI, we fit a series of models of individual BMI at time *t* on the block foreclosure rate in the prior year. All statistical analyses were performed with Stata 13. The general form of the model was specified as:

*Y*_*it*_ = *β*_*o*_ + *β*_1_*F*_*it*−1_ + *β*_2_*Z*_*it*−1_ + *β*_3_*C*_*it*_ + *β*_4_*X*_*i*_ + *β*_5_*yearD*_*t*_ + *u*_*i*_ + *v*_*it*_, where *Y*_*it*_ is a measure of the mean BMI level of individual *i* in year *t*; *F*_*it*−1_ is the block annual foreclosure rate for individual *i* in year *t-1*; *Z*_*it*−1_ is a vector of lagged area-level controls for individual *i* in year *t-1* (unemployment, housing prices); *C*_*it*_ is a vector of individual time-variant covariates including indicators for Medicaid status and medication use; *X*_*i*_ is a vector of individual time-invariant covariates such as sex and race; *yearD* are dummies for year *t*; *u*_*i*_ is an individual fixed-effect, and *v*_*it*_ is the time and individual specific error term.

Our primary parameter of interest, change in BMI for each one-unit change in the block foreclosure rate in the prior year, was estimated using an individual fixed effects approach by estimating a fixed-effect (*u*_*i*_) for each individual. The inclusion of individual fixed effects allowed us to control for observed and unobserved factors that do not change over time that might be correlated with both individual body mass index and neighborhood foreclosure rate such as credit scores and propensity to be present-biased.

As foreclosures and health status are not randomly distributed, our estimates might differ based on health insurance or demographic characteristics. First, we restricted our sample to those under 65 who used Medicaid at least once during the period (n = 4,463) and for the entire period (n = 3,138). Next, we stratified our results based on the individual’s baseline age and created three groups of adults (20–49, 50–64, 65+). Then we stratified by groups potentially differentially affected by the recession using race/ethnicity [45].

In order to test one of the mechanisms by which the community foreclosure rate is hypothesized to influence BMI, we stratified by the main fixed effects models by homeownership status (owners or renters) among the DISTANCE respondents who lived in the same address for the period. If homeowners experience a nearby foreclosure as a stressful event because it signals a future potential loss of housing wealth and the stress response provokes an increase in BMI, we expect the relationship between foreclosure rate and BMI to be greater among owners than renters [46].

### Sensitivity analyses

We conducted a series of sensitivity analyses to examine the consistency of the study results to different analysis specifications. First, we created two additional measures of foreclosure exposure and repeated the study analyses using these alternative definitions: a census block group foreclosure rate and a count of foreclosures within a 1-km Euclidean radius around the patient’s block centroid. Second, in order to facilitate comparisons to the single existing study on foreclosures and body mass index, we replicated the analytic approach used by Arcaya et al [20]. We used a linear mixed model fit with maximum likelihood containing both fixed and random effects with an independent covariance structure [47] to regress the lagged number of foreclosures in the patient’s census block on BMI, adjusting for education, income, age, race, sex, age, population density, foreclosures per 1km, and a quadratic time trend. Finally, we assessed the robustness of our main estimates to different lag structures, including two-year lag and contemporaneous specifications.

## Results

### Descriptive

There were 105,919 individuals in the study population that met inclusion criteria and had at least two measures of BMI between 2007 and 2010. Over half (58%) of the sample was clinically observed in all four years and 17.3% of the individuals moved at least once during the study period.

The means and standard deviations for the time-varying variables are reported in Table 1. The average BMI (kg/m^2^) in this cohort was 31.15 (s.d = 1.2 units) over the period. For a person 5’6” and 193 pounds, the standard deviation is equivalent to 7 pounds. There were 0.3 foreclosures in the average block (20 homes) and the within-person standard deviation was 0.6.

**Table 1:** Mean and standard deviation of within and between individual.

Variable	Mean	Standard Deviation		
		Overall	Between	Within
**Body Mass Index (BMI)**	31.15	7.02	6.97	1.23
**Block foreclosures per 20 homes**	0.28	0.85	0.70	0.58
**Block Group foreclosures per 2200 homes**	6.88	13.28	10.05	9.02
**Foreclosures per 1km**	26.03	43.22	26.46	34.93
**Mean Housing Prices per Zip-Code (US$)**	$537,699	$223,544	$200,504	$100,938
**County Unemployment Rate (%)**	6.2%	2.4%	0.9%	2.3%
**Charlson Comorbidity Index**	1.75	1.64	1.36	0.93
**Insulin Use**	13.8%	34.5%	30.5%	16.3%
**Oral Medication Use**	39.1%	48.8%	42.5%	24.3%
**Medicaid**	1.6%	12.5%	12.2%	3.9%

Table 1 shows the mean and the within and between individual standard deviation of each key variable.

Table 2 indicates substantive differences in baseline health, demographic and neighborhood characteristics between those whose blocks were least and most affected by the foreclosure crisis. Those who lived in blocks hit hardest by foreclosures during 2008 (i.e. top quintile) were more likely to be obese, Black, under 65, and living with a spouse or partner, while being far less likely to have a Bachelors degree, employment, and non-housing wealth of over $10,000 at baseline. These individuals also lived in neighborhoods at baseline with an average block foreclosure rate that was double that of the bottom quintile at baseline. The neighborhoods in the top quintile also had lower housing prices, more poverty, a higher rate of owner-occupied units, a lower proportion of Whites, and a lower population density.

**Table 2:** Baseline Characteristics of Participants and Their Neighborhoods, According to Exposure to Foreclosures in 2008.

	All Participants (n = 66,543)	Residence in bottom 5th foreclosures (n = 40,374)^a^	Residence in top 5th foreclosures (n = 13,089)
**Health characteristics**			
BMI (kg/m^2^)	31.1	30.7	31.9
Obese (BMI>30) (%)	49.7	47.6	55.2
**Demographic characteristics**			
Over 65 (%)	42.6	45.8	35.7
Non-Hispanic White (%)	48.9	52.0	40.1
Black (%)^b^	12.2	9.7	20.6
Asian (%)^b^	25.4	25.5	23.9
Income under $35k ^c^^,^^d^	27.7	27.3	27.9
Non-housing wealth (10,000+) (%)^a^	71.9	72.8	68.1
Homeowner (%)^c^	79.8	77.2	85.7
Employed (%)^c^	33.2	36.1	26.1
Bachelors degree + (%)^c^	32.3	35.3	24.2
Partnered (%)^c^	72.7	71.9	74.2
**Neighborhood characteristic**			
Block foreclosures per 1000 homes	1.4	1.2	2.4
Housing prices per zip-code	635,798.5	685,876	539,686.5
Percent owner-occupied in block group	66.1%	65.5%	67.5%
Percent poverty in block group	9.1%	8.3%	11.6%
Percent White in block group	54.2%	56.9%	47.9%
Population density per sq mile	9,780.3	10,252.9	8,556.9

Table 2 shows variables in total population, the least hardest hit blocks (bottom 5^th^), and the hardest hit blocks (top 5^th^). All census blocks in one of 9 Bay Area Counties were assigned to a quintile based on its foreclosure rate in 2008.

^a^ There was a larger number n = 40,374 of individuals living in the bottom quintile.

^b^ Black and Asian are categories for race and include Hispanic and non-Hispanic individuals; 23.5% of the total sample identify as Hispanic across all racial groups

^c^ Only available for the DISTANCE sub-set (n = 8,923).

^d^ There was a statistically significant difference (p<0.01) in the means of all variables between the top 5^th^ and bottom 5^th^, except for Income under $35k

### Main Results

The individual fixed effects models for the full cohort showed no statistically significant association between block foreclosure rate and BMI (Table 3). We observed a clinically irrelevant statistically significant relationship for the unadjusted model (Model 1), *β* = -0.007, 95% CI [-0.009, -0.005]. The addition of the year fixed effects attenuated the estimate (Model 2), *β* = 0.001, 95% CI [-0.001, 0.003], and coefficients on year indicators were negative, indicating that BMI decreased from 2007 to 2010. The model adjusting for unemployment rate and mean housing prices (Model 3) and the full set of individual time-varying covariates (Model 4) left the coefficient block foreclosure rate unchanged.

**Table 3:** Linear regression of block foreclosure rate on body mass index (BMI) within individual fixed effects.

	Model 1	Model 2	Model 3	Model 4
**Block foreclosures per 100 homes**_**(t -1)**_	-0.00674*** (0.001)	0.000909 (0.001)	0.00124 (0.001)	0.00123 (0.001)
**Unemployment rate**_**(t -1)**_			0.0304*** (0.012)	0.0270** (0.012)
**Mean Housing Price (logged)**_**(t -1)**_			0.0542** (0.024)	0.0556** (0.024)
**Medicaid**				0.108 (0.099)
**Charlson Comorbidity Index**				-0.0323*** (0.004)
**Insulin**				0.382*** (0.025)
**Oral Medication**				0.265*** (0.016)
**Weight Gain**				-0.0547 (0.037)
**Weight Loss**				-0.0775** (0.033)
**2008**		-0.0768*** (0.007)	-0.0822*** (0.008)	-0.0798*** (0.008)
**2009**		-0.151*** (0.009)	-0.184*** (0.021)	-0.179*** (0.021)
**2010**		-0.231*** (0.010)	-0.378*** (0.068)	-0.358*** (0.068)
**Intercept**	31.19*** (0.001)	31.29*** (0.005)	30.44*** (0.339)	30.32*** (0.342)

Table 3 shows point estimates (and clustered robust standard errors) for Models 1–4. There are 105,919 individuals and 331,917 observations in each model.

***p<0.01

**p<0.05.

For each one standard deviation increase in the county unemployment rate, individual mean BMI increased by a half pound on average (p<0.05). Decreases in mean housing prices had a statistically significant relationship association (p<0.05) with increased BMI, however the magnitude of the effect was less than a tenth of a pound gain for one a one standard deviation change in prices, and was too small to be clinically meaningful. Starting insulin or oral medication during the study period was associated with an increase of approximately two pounds (p<0.001).

### Subgroup-Specific Results

Table 4 contains results among Medicaid patients. Among those under 65 who were enrolled in Medicaid for at least one year during the period (Model 5, *β* = 0.004, 95% CI [-0.02, 0.03]) and for all four years (Model 6, *β* = 0.006, 95% CI [-0.01, 0.02]), there was no statistically significant effect of block foreclosure rate on BMI, contrary to our hypothesis.

**Table 4:** Linear regression of foreclosures on body mass index (BMI) with individual fixed effects for Medicaid Patients.

	(5)	(6)	(7)	(8)	(9)	(10)
	Block Foreclosure Rate		Block Group Foreclosure Rate		Foreclosures per 1km	
	1+ year	All years	1+ year	All years	1+ year	All years
**Foreclosure Measure**_**(t -1)**_	0.00428 (0.012)	0.006 (0.008)	-0.264** (0.105)	-0.098 (0.097)	0.0001 (0.001)	0.0005 (0.001)
**Unemployment rate**_**(t -1)**_	0.179 (0.191)	0.299* (0.168)	0.234 (0.191)	0.343** (0.165)	0.211 (0.192)	0.332** (0.170)
**Mean Housing Price (logged)**_**(t -1)**_	0.203 (0.247)	-0.004 (0.0232)	0.0544 (0.246)	-0.050 (0.234)	0.173 (0.262)	-0.001 (0.267)
**2008**	0.0454 (0.045)	-0.048 (0.100)	0.0741 (0.106)	-0.054 (0.099)	0.0405 (0.107)	-0.043 (0.104)
**2009**	-0.177 (0.352)	-0.498* (0.316)	-0.126 (0.356)	-0.522* (0.311)	-0.232 (0.354)	-0.560 (0.318)
**2010**	-1.012 (1.117)	-1.851* (0.987)	-1.299 (1.118)	-2.100** (0.971)	-1.212 (1.127)	-2.046** (1.010)
**Intercept**	31.64*** (3.453)	31.214*** (3.175)	33.34*** (3.431)	31.63*** (3.202)	31.83*** (3.600)	31.00*** (3.563)
**Individuals**	1469	1064	1469	1064	1469	1064
**Observations**	4463	3749	4463	3749	4463	3749

Table 4 shows point estimates (and clustered robust standard errors) for Models 5–10. ***p<0.01

**p<0.05

*p < 0.1.

S1 Table contains sub-group specific results stratified by age and race, while S2 Table provides contextual descriptive statistics by race. Estimates for patients over 50 years of age and for non-Hispanic Whites were similar to the full cohort, *β* = 0.001, 95% CI [-0.003, 0.004]. Among those ages 20 to 49 and among Asians, the coefficient on the foreclosure rate was negative and statistically insignificant. For Blacks, for a three percent (~ 1 standard deviation) increase in unemployment rate, there was a statistically significant (p<0.001) half unit increase in BMI or roughly three-pound gain for an average 5’6” and 193 pound person (*β* = 0.189, 95% CI [0.079, 0.300]). For those ages 20 to 49 and 65+, there was a statistically significant (p<0.001) change in BMI for every one standard deviation change in housing prices, however this effect was of a negligible magnitude. Among the DISTANCE cohort (not shown), there was no difference between the relationship between foreclosure rate and BMI among homeowners and renters.

### Sensitivity analysis

The main models were fit using alternate exposures, the block group foreclosure rate and the number of foreclosures within 1-km from the patient’s block centroid, and yielded similar results to the block foreclosure rate, *β* = 0.001, 95% CI [-0.001, 0.003] (not shown).

For those who used Medicaid at least once during the period, there was a statistically significant (p<0.05) decrease of 0.26 kg/m^2^ (~ 2 pounds) for every one percent increase in the block group foreclosure rate (Table 4, Model 7). We found no association between block group foreclosure rate and BMI among those who were enrolled in Medicaid for all four years (Model 8, *β* = -0.098, 95% CI [-0.287, 0.092]). There was no association between foreclosures per 1 km and BMI for those on Medicaid ever or for all four years (Models 9 and 10, 95% CI [-0.002, 0.003]).

The effect of unemployment on BMI among those who were observed to use Medicaid for all four years was robust across spatial measures of foreclosures; for every 3 percent (~ 1 standard deviation) increase in the unemployment rate, there was a statistically significant (p<0.05) increase of 1 kg/m^2^ (~ 7 pounds).

We observed no association between body mass index and a 2-year lagged block foreclosure rate or contemporaneous block foreclosure rate. Finally, the mixed effects model that replicated the study by Arcaya et al [20] yielded no statistically significant association between block foreclosure rate and body mass index (n = 57,791, *β* = -0.0001, 95% CI [-0.002, 0.002] (data not shown).

## Discussion

Among our continuously insured managed care population with diabetes, we found no evidence of an association between proximity to foreclosures in the prior year and BMI for the full cohort. Our results differ from the single existing study known on proximity to foreclosures and BMI [20] which found an additional foreclosure within 100 meters was associated with an increase of 0.2 units in BMI. Only 2% of the population (n = 2,068) in the study by Arcaya et al was exposed to at least one foreclosure at 100m, while more than half of our population was exposed to at least one foreclosure in the residential census block. Our population also included a much higher proportion of racial minorities (more than 50% compared to 0.5%). In our study, BMI was measured from visit level clinical data rather than self-reported measures and our foreclosure measure included all residential foreclosures instead of exclusively bank-owned foreclosures. Finally, our study focused on the recent residential foreclosure crisis (2006 to 2010) in California, rather than the time period 1987 to 2008 in Massachusetts included in Arcaya et al’s study.

Our results also differ from the findings by Currie and Tekin [17]; while both studies found no effect of the foreclosure crisis on health among those with private health insurance, their study showed an effect of a large magnitude among those with public health insurance. One reason for the difference might be the geographical variation in the timing of the Great Recession and the foreclosure crisis; while nationwide the foreclosure crisis preceded unemployment, the labor market declined before the housing market in more than half of metropolitan areas [48]. In the San Francisco-Oakland-Freemont Metropolitan area where a majority of our population resided, the housing market declined 7 quarters prior to the labor market. Currie and Tekin’s population included those in Arizona, California, New Jersey, and Florida [17]. These states contained metropolitan areas that have larger labor markets and, on average, experienced a shorter period between the decline of the housing and labor markets. It is possible that in some parts of these states, the labor market declined prior to or simultaneously with the foreclosure rate increase. The inclusion of lagged unemployment might have induced confounding and biased the results away from the null if the temporal ordering was misspecified.

In our study, among those under 65 who used Medicaid at least once over the period, there was a marginally significant negative association between block group foreclosure rate and BMI. The relationship did not hold for those who used Medicaid for all four years and thus our findings are consistent with research that has shown that ambulatory care-sensitive conditions such as diabetes are influenced by the interruption in Medicaid coverage [49].

Our study has several limitations. We were unable to measure job status and income, thus unmeasured residual confounding might persist. In addition, our data did not allow us to distinguish real estate owned foreclosures (REOs), which limits the comparability of our results with other studies on community-level health and health behaviors [16, 18–21, 26–28].

Our study only includes four years of observations which might not be long enough to detect an effect. Although we refer to the lag period as one year, it should be interpreted as an average; the actual lag period could have ranged from 1 to 23 months depending on the month of the foreclosure and the timing of clinical visits.

Our results might have limited external validity beyond diabetes patients within an integrated healthcare delivery system (Kaiser Permanente of Northern Californa). Finally, we are unable to observe those who stopped seeking healthcare, and thus our sample may be biased if those who did not return for visits had a differential exposure to neighborhood foreclosures. To address this, we compared those who were in the sample for all four years compared to the full cohort of all patients with more than two years of visits, and the estimates were similar.

Our study adds to the literature on foreclosure rates and health. Findings suggest that patients’ (physical) health did not “fall victim to the foreclosure epidemic” [50]. More research is needed to understand the impact of economic stressors on health outcomes across various populations including those with different models of healthcare delivery and financing.

## Supporting Information

S1 Table Sub-group specific linear regression of block foreclosure rate on body mass index (BMI) with individual fixed effects. S1 Table describes the association between the block foreclosure rate and body mass index within age and race specific groups.
(DOCX)

S2 Table Mean and standard deviation of within and between individual, by Race. S2 Table presents the mean and standard deviations of key variables in each of the three most predominant race groups.
(DOCX)
