# Supplementary material for: No Spillover Effect of the Foreclosure Crisis on Weight Change: The Diabetes Study of Northern California (DISTANCE)
Source: PLoS One. 2016 Mar 17;11(3):e0151334. doi: 10.1371/journal.pone.0151334 (PMC4795787; doi:10.1371/journal.pone.0151334)
Supplement: S1 Table — describes the association between the block foreclosure rate and body mass index within age and race specific groups. (DOCX) [file pone.0151334.s001.docx]

| **S1 Table: Sub-group specific linear regression of block foreclosure rate on body mass index (BMI) with individual fixed effects** | | | | | | |
| --- | --- | --- | --- | --- | --- | --- |
|  | (A1) | (A2) | (A3) | (A4) | (A5) | (A6) |
| Variables | Age 20-49 | Age 50-64 | Age 65+ | Black | Non-Hispanic White | Asian |
| Block foreclosures per 100 homes _(t -1)_ | -0.003 | 0.002 | 0.001 | 0.004 | 0.001 | -0.001 |
|  | (0.003) | (0.002) | (0.002) | (0.002) | (0.002) | (0.001) |
| Unemployment rate _(t -1)_ | 0.082** | 0.030 | 0.019 | 0.189*** | 0.030 | 0.014 |
|  | (0.042) | (0.022) | (0.018) | (0.056) | (0.021) | (0.019) |
| Mean Housing Price (logged) _(t -1)_ | 0.251*** | -0.021 | 0.124*** | 0.130 | 0.064 | 0.023 |
|  | (0.073) | (0.045) | (0.035) | (0.072) | (0.046) | (0.023) |
| Medicaid | -0.114 | 0.203 | 0.173 | 0.101 | 0.048 | 0.116 |
|  | (0.246) | (0.163) | (0.147) | (0.324) | (0.225) | (0.161) |
| Charlson Comorbidity Index | 0.014 | -0.022** | -0.037*** | -0.032** | -0.052*** | -0.018** |
|  | (0.014) | (0.008) | (0.006) | (0.014) | (0.006) | (0.008) |
| Insulin | 0.529*** | 0.383*** | 0.304*** | 0.361*** | 0.423*** | 0.299*** |
|  | (0.070) | (0.038) | (0.040) | (0.077) | (0.041) | (0.044) |
| Oral Medication | 0.190*** | 0.217*** | 0.325*** | 0.115** | 0.326*** | 0.209*** |
|  | (0.038) | (0.027) | (0.027) | (0.052) | (0.029) | (0.028) |
| Weight Gain | 0.0213 | 0.009 | -0.133** | -0.048 | -0.075 | -0.150 |
|  | (0.126) | (0.061) | (0.053) | (0.115) | (0.054) | (0.094) |
| Weight Loss | -0.040 | 0.0237 | -0.175*** | -0.169 | -0.0677 | -0.224** |
|  | (0.086) | (0.061) | (0.046) | (0.110) | (0.044) | (0.077) |
| 2008 | -0.059** | -0.035*** | -0.134*** | -0.131*** | -0.103*** | -0.037*** |
|  | (0.026) | (0.013) | (0.011) | (0.030) | (0.013) | (0.013) |
| 2009 | -0.160** | -0.142*** | -0.230*** | -0.451*** | -0.228*** | -0.075** |
|  | (0.077) | (0.037) | (0.029) | (0.093) | (0.037) | (0.033) |
| 2010 | -0.519** | -0.300** | -0.412*** | -1.337*** | -0.435*** | -0.149 |
|  | (0.242) | (0.124) | (0.098) | (0.310) | (0.118) | (0.109) |
| Intercept | 29.89*** | 32.16*** | 27.72*** | 30.69*** | 31.47*** | 26.77*** |
|  | (1.019) | (0.634) | (0.489) | (1.064) | (0.640) | (0.587) |
| Individuals | 18534 | 41800 | 39712 | 10595 | 40891 | 22782 |
| Observations | 45888 | 118284 | 135662 | 32919 | 130455 | 70255 |

S1 Table describes the association between the block foreclosure rate and body mass index within age and race specific groups. Robust Standard errors are in parenthesis. ***p<0.01, **p<0.05, *p < 0.1
