# Supplementary material for: No Spillover Effect of the Foreclosure Crisis on Weight Change: The Diabetes Study of Northern California (DISTANCE)
Source: PLoS One. 2016 Mar 17;11(3):e0151334. doi: 10.1371/journal.pone.0151334 (PMC4795787; doi:10.1371/journal.pone.0151334)
Supplement: S2 Table — presents the mean and standard deviations of key variables in each of the three most predominant race groups. (DOCX) [file pone.0151334.s002.docx]

| **Table S2: Mean and standard deviation of within and between individual, by Race** | | | | | | | | | | | | |
| --- | --- | --- | --- | --- | --- | --- | --- | --- | --- | --- | --- | --- |
|  | **Black** | | | | **Asian** | | | | **Non-Hispanic White** | | | |
| Variable | Mean | Standard Deviation | | | Mean | Standard Deviation | | | Mean | Standard Deviation | | |
|  |  | Overall | Between | Within |  | Overall | Between | Within |  | Overall | Between | Within |
| *Body Mass Index (BMI)* | 33.14 | 7.58 | 7.56 | 1.37 | 27.20 | 4.86 | 4.78 | 0.93 | 32.37 | 7.20 | 7.17 | 1.34 |
| *Block Foreclosure Rate per 100 homes* | 1.98 | 4.70 | 3.38 | 3.44 | 1.44 | 4.93 | 4.42 | 3.27 | 1.06 | 3.50 | 2.95 | 2.42 |
| *Zip-Code Mean Housing Prices (thousands US$)* | $436.3 | $192.6 | $160.1 | $110.4 | $570.9 | $222.5 | $201.0 | $97.3 | $562.5 | $236.8 | $216.9 | $97.0 |
| *County Unemployment Rate (%)* | 6.4% | 2.4% | 0.9% | 2.3% | 6.2% | 2.4% | 0.9% | 2.3% | 6.2% | 2.4% | 0.9% | 2.3% |
| *Charlson Co-Morbidity Index* | 1.89 | 1.73 | 1.44 | 0.98 | 1.55 | 1.46 | 1.19 | 0.86 | 1.92 | 1.75 | 1.45 | 1.00 |
| *Insulin Use* | 16.0% | 36.6% | 32.2% | 17.6% | 9.0% | 28.6% | 24.9% | 14.2% | 16.3% | 37.0% | 33.0% | 16.9% |
| *Oral Medication Use* | 39.9% | 49.0% | 42.5% | 24.5% | 41.5% | 49.3% | 43.2% | 24.0% | 37.6% | 48.4% | 42.6% | 23.4% |
| *Medicaid* | 3.0% | 17.1% | 17.0% | 4.7% | 1.8% | 13.2% | 12.8% | 3.8% | 0.9% | 9.3% | 9.2% | 3.0% |

Table S2 presents the mean and standard deviations of key variables in each of the three most predominant race groups.
